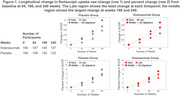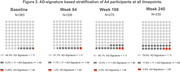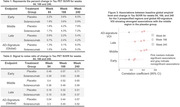# Longitudinal Regional Flortaucipir Profiles in Preclinical Alzheimer’s Disease: Evaluation using A4 phase 3 trial with Solanezumab

**DOI:** 10.1002/alz.093920

**Published:** 2025-01-09

**Authors:** Vikas Kotari, Karen Chilcott Holdridge, Roy Yaari, Aaron P Schultz, Keith A Johnson, Paul S. S. Aisen, Reisa A Sperling, John R. Sims, Sergey Shcherbinin

**Affiliations:** ^1^ Eli Lilly and Company, Indianapolis, IN USA; ^2^ Center for Alzheimer Research and Treatment, Brigham and Women’s Hospital, Massachusetts General Hospital, Harvard Medical School, Boston, MA USA; ^3^ Departments of Neurology and Radiology, Massachusetts General Hospital, Harvard Medical School, Boston, MA USA; ^4^ Alzheimer’s Therapeutic Research Institute, Keck School of Medicine, University of Southern California, San Diego, CA USA

## Abstract

**Background:**

The A4 Study (NCT02008357) was a multicenter, randomized, double‐blind, placebo‐controlled phase 3 trial, assessing the safety and efficacy of solanezumab in preclinical Alzheimer’s Disease (AD). We previously reported on the baseline regional flortaucipir (FTP) profiles in this study1. Here, we present the longitudinal tau profiles.

**Methods:**

FTP scans were acquired at baseline and weeks 84,168, and 240. All FTP scans were analyzed using an established image processing pipeline to evaluate global[2] and regional tau patterns in template space. Global tau level was measured using an AD‐signature weighted volume‐of‐interest (VOI) (tauSUVR)2. Regional tau uptake and accumulation were measured in 3 prespecified composite regions representing tau pathology in preclinical AD, ordered by hypothetical pathologic spreading: 1) “early” (amygdala, entorhinal cortex, parahippocampal gyrus), 2) “middle” (fusiform, inferior, and middle temporal, and inferior parietal gyri), and 3) “late” (lateral occipital, posterior cingulate, superior parietal, and supramarginal gyri). We evaluated associations between baseline amyloid burden, age, sex, APOE e4 carriage and longitudinal tau change within placebo group.

**Results:**

At baseline, a lower tau signal was observed generally in regions identified later in pathologic sequences. Longitudinal change was region‐ and visit‐specific. In both treatment groups, the early and middle regions showed the largest change from baseline at Week 84; the middle region showed the largest change at Weeks 168 and 240; the global assessment changes were smaller than the early and middle regions at each time point (figure 1; tables 1&2) and a majority of participants remained global “tau negative”3(tauSUVR<1.11 figure 2) throughout the trial. Higher baseline global amyloid level was most strongly associated with increased tau PET signal in the prespecified middle composite region at all timepoints (figure 3).

**Conclusion:**

A multi‐regional composite VOI staging scheme was developed using baseline tau profiles and prespecified for analysis of longitudinal tau PET images. Results confirmed that cognitively unimpaired, amyloid positive participants demonstrate larger tau accumulation in the early and middle than late and neocortical regions. The early composite performed better at detecting tau accumulation initially and middle composite at later timepoints. This multi‐regional staging scheme appears to be useful to characterize tau progression in this preclinical AD cohort.